# *QuickStats:* Death Rates* Attributed to Excessive Cold or Hypothermia**^†^** Among Persons Aged ≥15 Years, by Urban-Rural Status**^§^** and Age Group — National Vital Statistics System, United States, 2019

**DOI:** 10.15585/mmwr.mm7007a6

**Published:** 2021-02-19

**Figure:**
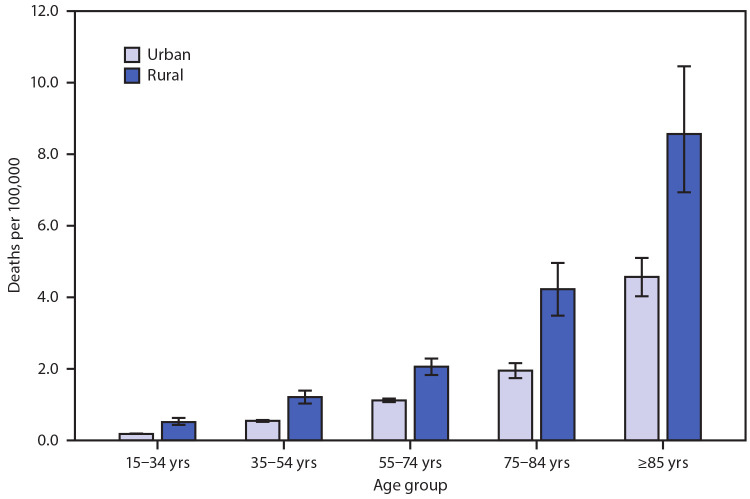
In 2019, among persons aged ≥15 years, death rates attributed to excessive cold or hypothermia were higher in rural areas than in urban areas across every age group. Crude rates were lowest among those aged 15–34 years at 0.2 and 0.5 per 100,000 population in urban and rural areas, respectively. Rates increased with age, with the highest rates among those aged ≥85 years at 4.6 in urban areas and 8.6 in rural areas. Differences between urban and rural rates also increased with age.

**Source:** National Center for Health Statistics, National Vital Statistics System, Mortality Data 2019. https://wonder.cdc.gov/mcd-icd10.html

